# Primary vitrectomy for degenerative and tractional lamellar macular holes: A systematic review and meta-analysis

**DOI:** 10.1371/journal.pone.0246667

**Published:** 2021-03-05

**Authors:** Guglielmo Parisi, Matteo Fallico, Andrea Maugeri, Martina Barchitta, Antonella Agodi, Andrea Russo, Antonio Longo, Teresio Avitabile, Niccolò Castellino, Vincenza Bonfiglio, Roberto Dell’Omo, Claudio Furino, Gilda Cennamo, Robert Rejdak, Katarzyna Nowomiejska, Mario Toro, Paola Marolo, Luca Ventre, Michele Reibaldi

**Affiliations:** 1 Department of Ophthalmology, University of Catania, Catania, Italy; 2 Department of Medical and Surgical Sciences and Advanced Technologies “GF Ingrassia”, University of Catania, Catania, Italy; 3 Department of Experimental Biomedicine and Clinical Neuroscience, Ophthalmology Section, University of Palermo, Palermo, Italy; 4 Department of Medicine and Health Sciences “V. Tiberio”, University of Molise, Campobasso, Italy; 5 Department of Ophthalmology, University of Bari, Bari, Italy; 6 Department of Public Health, University of Naples Federico II, Naples, Italy; 7 Department of General Ophthalmology, Medical University of Lublin, Lublin, Poland; 8 Department of Surgical Sciences, Eye Clinic Section, University of Turin, Turin, Italy; National Yang-Ming University Hospital, TAIWAN

## Abstract

**Purpose:**

To assess the efficacy of vitrectomy in degenerative and tractional lamellar macular holes (LMHs) by meta-analysis of published studies.

**Methods:**

PubMed, Medline and Embase databases were searched up to May 2020. Included cohorts were divided into three groups: degenerative LMH group, lamellar hole associated epiretinal proliferation (LHEP) group and tractional LMH group. LHEP is likely to be associated with degenerative LMHs, but less commonly could be associated with mixed LMHs. To reduce risk of possible misclassification bias, eyes with LHEP which could not have been precisely classified by the authors, were included into the LHEP group. The primary outcome was to investigate the visual change following primary vitrectomy in the degenerative LMH and LHEP group versus the tractional LMH group. A sensitivity analysis excluding the LHEP group was also performed on the primary outcome. Mean difference (MD) in best corrected visual acuity between baseline and post-treatment was calculated, along with 95% confidence interval (CI). Rate of incidence of post-operative full-thickness macular hole (FTMH) was assessed as secondary outcome.

**Results:**

Thirteen studies were included. Pooled analyses including all groups showed a significant visual improvement following vitrectomy (pre-post MD = -0.17;95%CI = -0.22,-0.12;p<0.001), with no difference in visual improvement between the degenerative LMH and LHEP group and the tractional LMH group. The sensitivity analysis excluding LHEP group confirmed no difference in visual change between the degenerative LMH group (pre-post MD = -0.18;95%CI = -0.24,-0.12;p<0.001) and the tractional LMH group (MD = -0.16;95%CI = -0.26,-0.07;p<0.001). The incidence rate of post-operative FTMH was higher in the degenerative LMH and LHEP group than in the tractional LMH group (p = 0.002).

**Conclusion:**

Primary vitrectomy for LMH ensured a favorable visual outcome, with no difference in visual gain between degenerative and tractional LMHs. However, a higher incidence of post-operative FTMHs was found in eyes with the degenerative LMH subtype.

## Introduction

Since the first optical coherence tomography (OCT)-based description was published in 1998, the definition of lamellar macular hole (LMH) has continued to be refined [1]. This is because major advances in OCT technology have significantly improved the ability to study the foveal contour, the integrity of the outer retinal layers and the epiretinal materials associated with LMH. Moreover, histopathologic analyses have clarified the cellular composition of the different epiretinal materials that can be visualized in association with LMH using OCT [2].

In 2016, Govetto and coworkers proposed classifying LMHs into two subtypes, which are characterized by different pathogenetic and clinical features: degenerative LMH and tractional LMH [3]. Distinctive features of the former one are the presence of lamellar hole-associated epiretinal proliferation (LHEP), foveal bump and, in most cases, an ellipsoid disruption. The latter one’s main characteristic is the presence of a tractional epiretinal membrane [3]. This classification has become a landmark in this field, gaining significant clinical relevance when it comes to the management of the two LMH subtypes.

In particular, the issue whether vitreoretinal surgery could be beneficial for both tractional and degenerative subtypes is still controversial. Some studies reported a visual improvement following treatment of both subtypes [4, 5], while others described an increase in visual function only for tractional LMHs [6, 7]. Additionally, degenerative LMHs seemed to be associated with a higher incidence of full-thickness macular hole (FTMH) following surgery [2, 6].

On this basis, we systematically reviewed the literature and performed a meta-analysis with the purpose of comparing visual and anatomical outcomes of pars plana vitrectomy between tractional and degenerative LMHs.

## Materials and methods

### Literature search methods

The study was conducted according to the guidelines of the Preferred Reporting Items for Systematic Reviews and Meta-Analyses (PRISMA) group (PRISMA checklist available in S1 Table as supplementary material) [8].

We conducted comprehensive searches of PubMed, Medline and Embase databases. The electronic search strategy included the terms “lamellar macular hole”, “lamellar macular holes”, “vitrectomy”, “degenerative”, “tractional” and “pars plana vitrectomy” in various combinations. The last search was done on May 25, 2020. Only publications in peer-reviewed journals and in English were considered. No publication date or publication status restrictions were imposed. When needed, we contacted the authors of the relevant publications for clarification on eligibility assessment.

### Eligibility criteria

Included studies had to meet the following eligibility criteria: 1) to include tractional LMHs and/or degenerative LMHs that were treated with primary vitrectomy; 2) to report the primary outcome of interest of this systematic review; 3) to differentiate visual outcome within each LMH subtype. Case reports and case series with less than 10 cases were excluded. Idiopathic full-thickness macular holes and myopic macular holes were excluded.

Tractional LMHs and degenerative LMHs were defined by the authors within each eligible study. If the subtype of hole was not specifically defined by the author within the study, the presence of specific diagnostic criteria was sought within the study according to the diagnostic criteria of Govetto *et al*. [3]. In particular, the presence of conventional tractional epiretinal membrane (ERM) was mandatory for a LMH to be classified as tractional. Tracional LMHs were included in the tractional LMH group. With regards to those studies that enrolled eyes with LHEP, the authors were contacted to ascertain whether these cases could be classified as degenerative according to Govetto’s study [3]. Author of three studies [2, 5, 9] confirmed that their cases with LHEP can be classified as degenerative. Degenerative LMHs were included in the degenerative LMH group. Those studies including LHEP eyes whose authors were unable to give information on the precise subtype of the LMH, were considered as the LHEP group [7, 10–13].

The primary outcome was to investigate the visual change following primary vitrectomy including eyes with degenerative LMHs and LHEP (degenerative LMH group and LHEP group) on the one side and eyes with tractional lamellar macular hole on the other side (tractional LMH group). A sensitivity analysis excluding the LHEP group was also performed on the primary outcome.

Incidence rate of full-thickness macular holes (FTMH) after primary vitrectomy was considered a secondary outcome measure as well as meta-regression analyses performed to study the influence of the following variables on the primary outcome: mean age, follow-up duration and baseline best corrected visual acuity (BCVA).

### Data collection and risk of bias assessment

The eligibility of patient cohorts was assessed independently by two senior investigators (G.P. and A.R.). Disagreements between investigators were resolved by discussion involving a third investigator. All article information, including outcomes, was captured in the data extraction and assessment of risk of bias form. We used a standard form to extract data; this data extraction and assessment of risk of bias form was used as the source of outcome data and to assess the potential for bias in the design or execution of each study.

The following additional information was extracted from all included studies: names of the first author, year of publication, study design, median year when the study was performed, average patient age, type of surgical technique, ellipsoid zone baseline integrity, presence of LHEP, presence of contractile ERM, and mean follow-up duration.

We contacted the authors of studies to request additional information whenever the article did not report the variables we wished to analyze.

Study quality was assessed by two investigators (G.P. and A.R.) using the Newcastle–Ottawa Quality Assessment Scale (NOS), which evaluates patient selection, comparability of degenerative LMH group and tractional LMH group, and outcome [14]. The risk of bias for each included study was categorized as high when the NOS score was < 6, and low-to-moderate when ≥6 [15]. Publication bias was evaluated by examination of the funnel plot for evidence of asymmetry and the Egger regression test.

### Data synthesis and analysis

The primary outcome was the mean difference in BCVA between baseline and post-treatment (i.e. pre-post mean difference, MD) with 95% Confidence Interval (95%CI). For those articles investigating both degenerative and tractional lamellar macular holes, we assessed pre-post mean differences separately. Heterogeneity across studies was tested using the Q-statistics, while its degree was measured using the I^2^ index. In the presence of significant heterogeneity (p-value for Q-statistics <0.01 and I^2^ > 50%), a random effect model with the DerSimonian-Laird method was applied. To compare the effect of vitrectomy on BCVA in the different groups, a subgroup analysis was carried out. Furthermore, random-effect meta-regressions were used to test the impact of moderators (i.e. mean age, follow-up duration, proportion of patients with LHEP, and baseline BCVA) on the effect size. We also carried out a sensitivity analysis by excluding studies classified into the LHEP group and studies with modified peeling techniques. The extent of publication bias was explored by funnel plots and tested using Egger’s test.

We also examined the incidence rate of FTMH as secondary outcome, both overall and stratified for subtype of lamellar macular hole. Specifically, the score confidence intervals were constructed for each individual study and incidence rates were pooled using the random-effects model [16]. Statistical analyses were performed using STATA (version 16). All the analyses were two-tailed, with a significance level of α < 0.05, if not otherwise stated.

## Results

### Selection of studies

**Fig 1:**
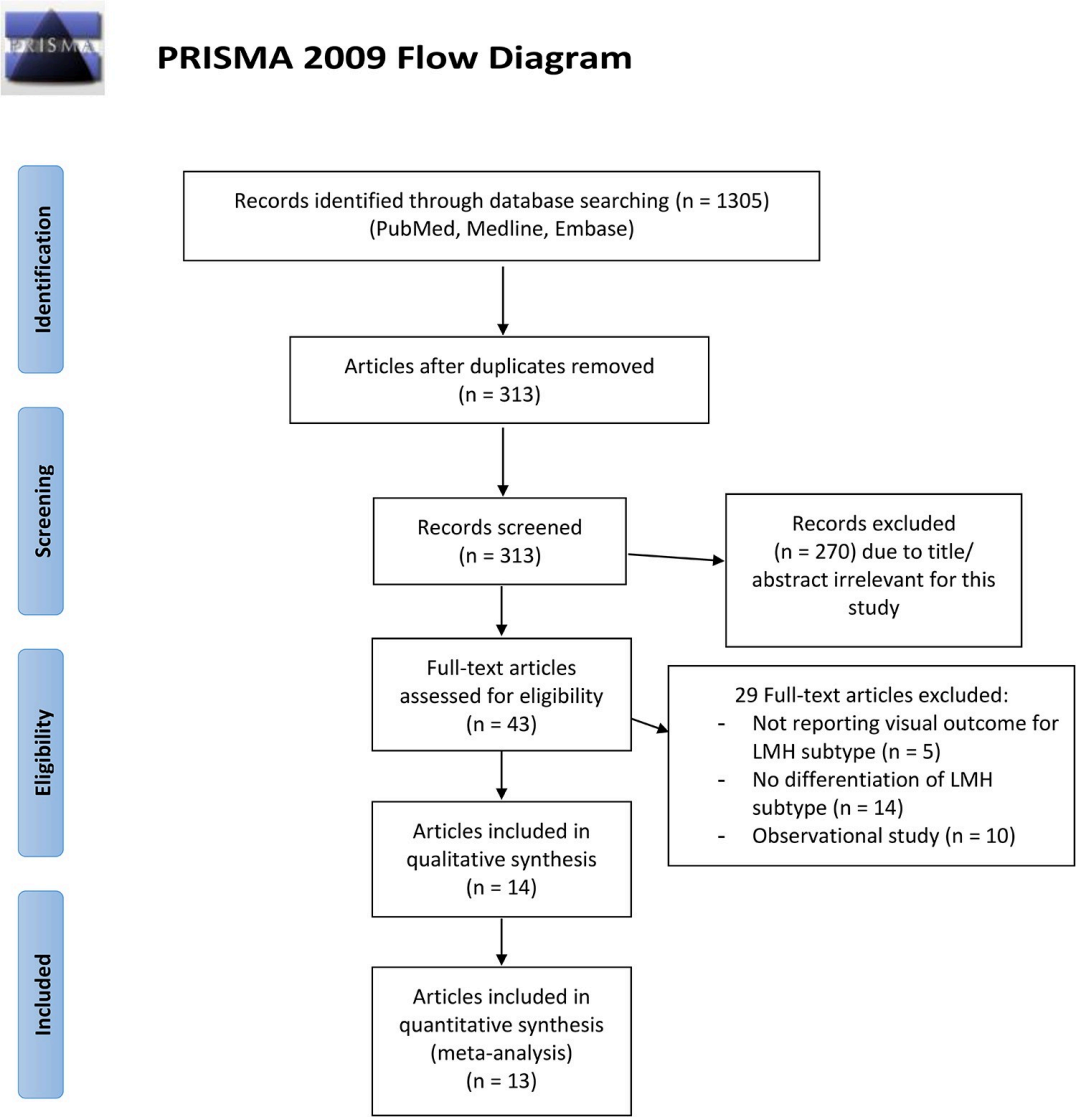
Flow chart of study selection process. *From*: Moher D, Liberati A, Tetzlaff J, Altman DG, The PRISMA Group (2009). *P*referred *R*eporting *I*terns for *S*ystematic Reviews and *M*eta-*A*nalyses: The PRISMA Statement. PLoS Med 6(7): e1000097. doi: 10.1371/journal.pmed1000097 **For more information, visit** www.prisma-statement.org.

The study selection process is shown in Fig 1. A total of 1305 studies were identified from the electronic database search, of which 992 were duplicates. The remaining 313 articles were screened by applying inclusion and exclusion criteria, and 43 potentially relevant studies were identified. Twenty-nine studies were ruled out after full-text assessment. A total of 14 studies were included in this systematic review, of which 13 were pooled together for the quantitative analysis, while one study was excluded from the analysis being the only randomized clinical trial.

### Study characteristics

Of the 14 studies included in this systematic review, only one featured a randomized design, while 13 were retrospective studies. The randomized clinical trial was conducted by Morescalchi *et al*. [17] and compared a foveal sparing internal limiting membrane (ILM) peeling to only observation in 34 patients affected by degenerative lamellar macular hole with LHEP. This trial showed a better visual outcome over a 6-month follow-up in the group treated with vitrectomy and foveal sparing ILM peeling. The study of Morescalchi *et al*. was excluded from the quantitative analysis being the only one featuring a randomized design.

Meta-analyses were conducted pooling together data from the 13 retrospective studies. These studies were published in years between 2011 and 2019. Overall, 8 studies provided data on surgical outcomes of patients with degenerative lamellar macular holes [2, 4–6, 9, 18–20]; 6 out of the 13 studies reported tractional LMHs [2, 4–6, 18, 19]; 5 out of the 13 studies reported outcomes of eyes with LHEP [7, 10–13]. A total of 463 eyes were included in the pooled analyses, of which 176 were in the degenerative LHM group, 91 in the LHEP group and 196 in the tractional LMH group (Table 1). The mean follow-up was 22.19 months in the degenerative LMH group, 18.52 months in the LHEP group and 23.58 months in the tractional LMH group.

**Table 1:** Characteristics and grouping of included studies.

	STUDY	NUMBER OF EYES	LHEP	ERM	IS/OS DEFECT AT BASELINE	FTMH AFTER PPV
**DEGENERATIVE LMH**	*Parolini et al*. *2011*	13	9/13	nr	9/13	3
	*Choi et al*. *2017*	11	11/11	0/11	10/11	3
	*Coassin et al*. *2017*	19	11/19	nr	nr	3
	*Dell’Omo et al*. *2017*	12	12/12	8/12	8/12	3
	*Figueroa et al*. *2018*	26	26/26	0	13/26	2
	*Frisina et al*. *2018*	48	48/48	nr	20/48	3
	*Obata et al*. *2019*	13	9/13	nr	7/13	0
	*Takahashi et al*. *2019*	34	34/34	nr	15/34	0
**LMH WITH LHEP**	*Lai et al*. *2015*	19	15/19	nr	13/19	nr
	*Compera et al*. *2015*	10	10/10	nr	8/10	0
	*Ko et al*. *2016*	15	15/15	nr	8/15	0
	*Lai et al*. *2017*	14	14/14	nr	8/14	nr
	*Ho et al*.*2019*	33	33/33	0/33	10/33	0
**TRACTIONAL LMH**	*Parolini et al*. *2011*	6	0/6	6/6	0/6	0
	*Choi et al*. *2017*	11	0/11	11/11	3/11	0
	*Coassin et al*. *2017*	69	nr	69/69	nr	0
	*Dell’Omo et al*. *2017*	14	0/14	14/14	2/14	0
	*Figueroa et al*. *2018*	77	1/77	77/77	11/77	1
	*Obata et al*. *2019*	19	3/19	19/19	3/19	0

LMH, lamellar macular hole; LHEP, lamellar hole associated epiretinal proliferation; ERM, epiretinal membrane; FTMH, full thickness macular hole; IS/OS, inner segment/ outer segment; nr, not reported.

In the degenerative LMH group, the presence of LHEP was identified in 164/176 eyes (93.2%), in the tractional LMH group, the presence of LHEP was identified in 4/196 eyes (2%); a contractile epiretinal membrane was found in 8/176 eyes (4.5%) in the degenerative LMH group and in 196/196 eyes (100%) in the tractional LMH group; ellipsoid defect was found in 82/176 (46.6%) eyes in the degenerative LMH group and in 19/196 (9.7%) eyes in the tractional LMH group (Table 1).

All 463 eyes underwent vitrectomy. In 10 studies [2, 4–7, 9–13, 18, 19] conventional vitrectomy with ERM and ILM peeling was performed, while in 3 studies vitrectomy was performed along with a modified peeling technique such as the double inverted flap technique described by Frisina *et al*. [9], the LHEP-embedding technique described by Takahashi *et al*. [20] and LHEP-embedding combined with foveal ILM non-peeling described by Ho *et al*. [12].

### Quality assessment and risk of bias

S2 Table (available as supplementary material) shows the quality score of the 13 retrospective studies according to the NOS. All studies had a quality score ≥ 6, showing a low-to-moderate risk of bias. All studies were given 3 stars out of 4 for selection category and one star out of 2 for Confounder category. For the exposure category, six studies scored 3 stars out of 4 and seven studies obtained 2 stars. The symmetry of the funnel plot was evaluated to investigate the possible extent of publication biases, overall and stratified by LMH subtype. In the whole analysis, we obtained a nearly symmetrical funnel plot as confirmed by Egger’s test (p = 0.314; S1 Fig). Similarly, the funnel plots for different LMH subtypes revealed no evidence of publication bias, featuring a nearly symmetrical shape (p = 0.856 and p = 0.468, S2 Fig).

### Visual outcome

First, a comparison between the degenerative LMH and LHEP group versus the tractional LMH group was conducted, showing no statistically significant difference in baseline BCVA (mean = 0.47; 95%CI = 0.35, 0.60 for degenerative LMH and LHEP group vs. mean = 0.38; 95%CI = 0.24, 0.52 for tractional LMHs; p = 0.350).

**Fig 2:**
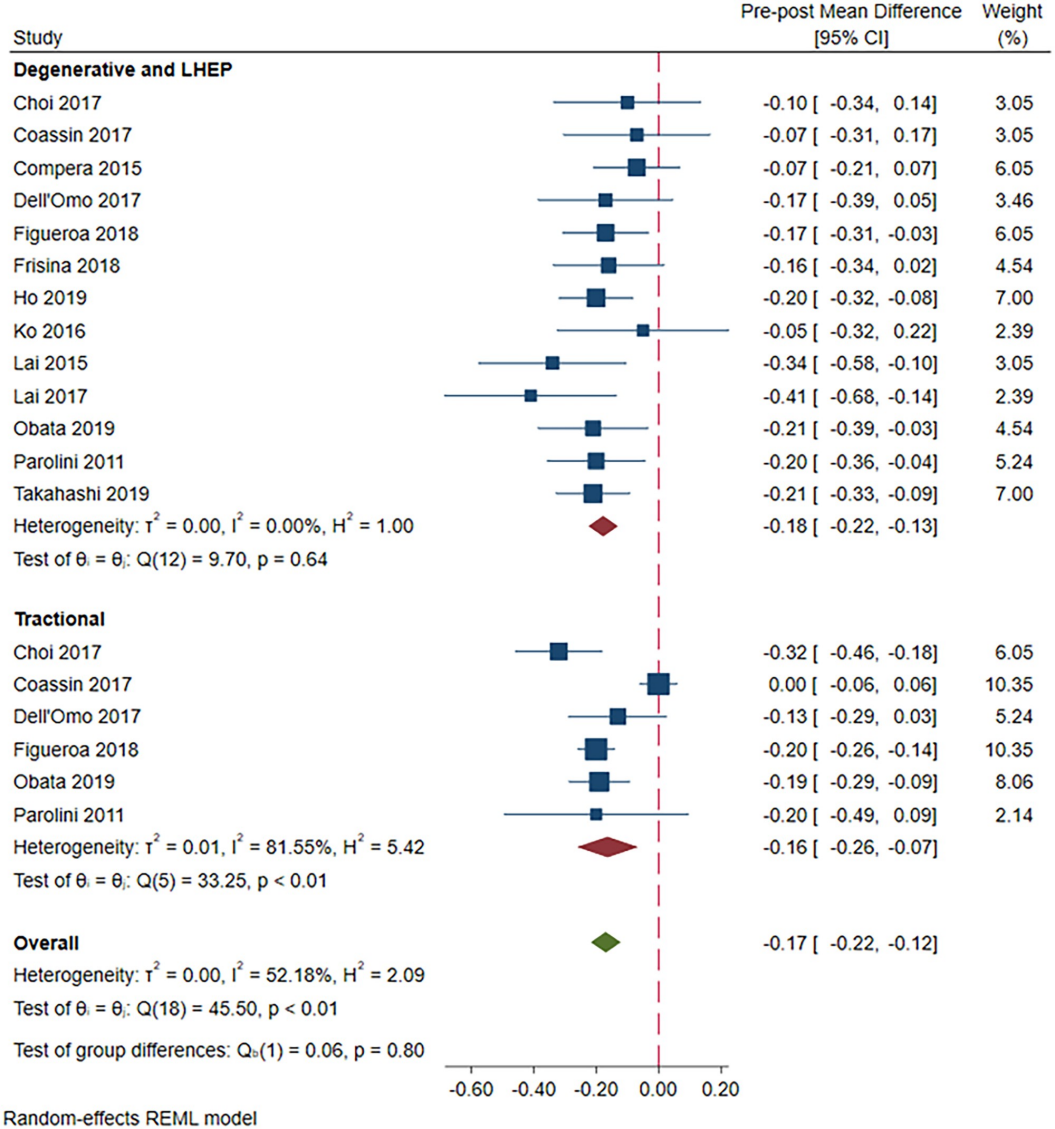
A forest plot showing pre-post mean difference in BCVA in the degenerative LMH and LHEP group versus the tractional LMH group.

Vitrectomy yielded a significantly improved postoperative BCVA irrespective of lamellar macular hole subtype (pre-post MD = -0.17; 95%CI = -0.22, -0.12; p<0.001; Fig 2). However, the Q-statistics and I^2^ indicated significant heterogeneity across studies (p<0.01 for Q-statistics and I^2^ = 52.2%). Subgroup analysis demonstrated no difference in post-operative BCVA improvement, when studies were evaluated separately by lamellar macular hole subtype. Indeed, post-operative BCVA significantly improved both in patients with degenerative LMHs and LHEP (MD = -0.18; 95%CI = -0.22, -0.13; p<0.001; Fig 2) and in patients with tractional LMHs (MD = -0.16; 95%CI = -0.26, -0.07; p<0.001; Fig 2). While heterogeneity disappeared across studies on patients with degenerative LMH and LHEP (p = 0.64 for Q-statistics and I^2^ = 0%), however, it remained high among those with tractional LMH (p<0.01 for Q-statistics and I^2^ = 81.6%).

**Fig 3:**
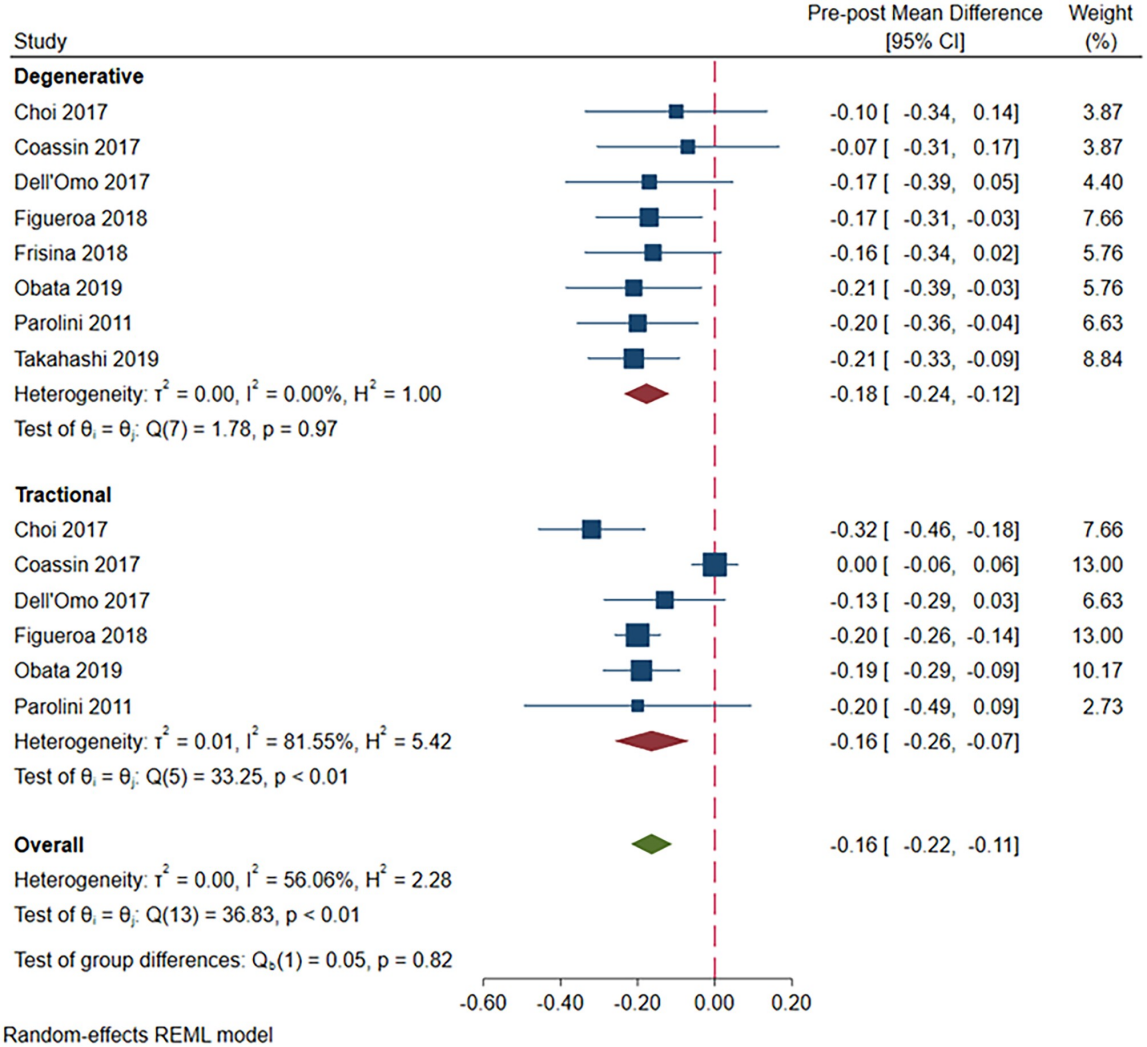
A forest plot showing pre-post mean difference in BCVA in the degenerative LMH group versus the tractional LMH group.

These findings were confirmed by a sensitivity analysis that excluded the LHEP group. In the degenerative LMH group, postoperative BCVA was significantly better compared with baseline values (pre-post MD = -0.18; 95% CI = -0.24, -0.12; p<0.001), and no heterogeneity was found (I^2^ = 0%, P = 0.97). In the tractional LMH group, postoperative BCVA was significantly better compared with baseline values (MD = -0.16; 95%CI = -0.26, -0.07; p<0.001; Fig 3), but significant heterogeneity was found (p<0.01 for Q-statistics and I^2^ = 81.6%). No difference in postoperative BCVA improvement was found between the two groups (overall pre-post MD = -0.16; 95% CI = -0.22, -0.11).

A further sensitivity analysis which excluded patients undergoing a surgically modified peeling technique was conducted (S3 Fig). We observed that BCVA significantly improved after vitrectomy in the whole analysis (pre-post MD = -0.15; 95%CI = -0.21, -0.10; p<0.001), with no significant difference between the degenerative LMH and LHEP group (MD = -0.14; 95%CI = -0.20, -0.09; p<0.001) and the tractional LMH group (MD = -0.16; 95%CI = -0.26, -0.07; p<0.001). However, the Q-statistics and I^2^ indicated significant heterogeneity across studies in the whole analysis (p<0.01 for Q-statistics and I^2^ = 55.5%) and in the subgroup of tractional LMH (p<0.01 for Q-statistics and I^2^ = 81.6%).

### The effect of moderators on post-operative visual acuity improvement

**Fig 4:**
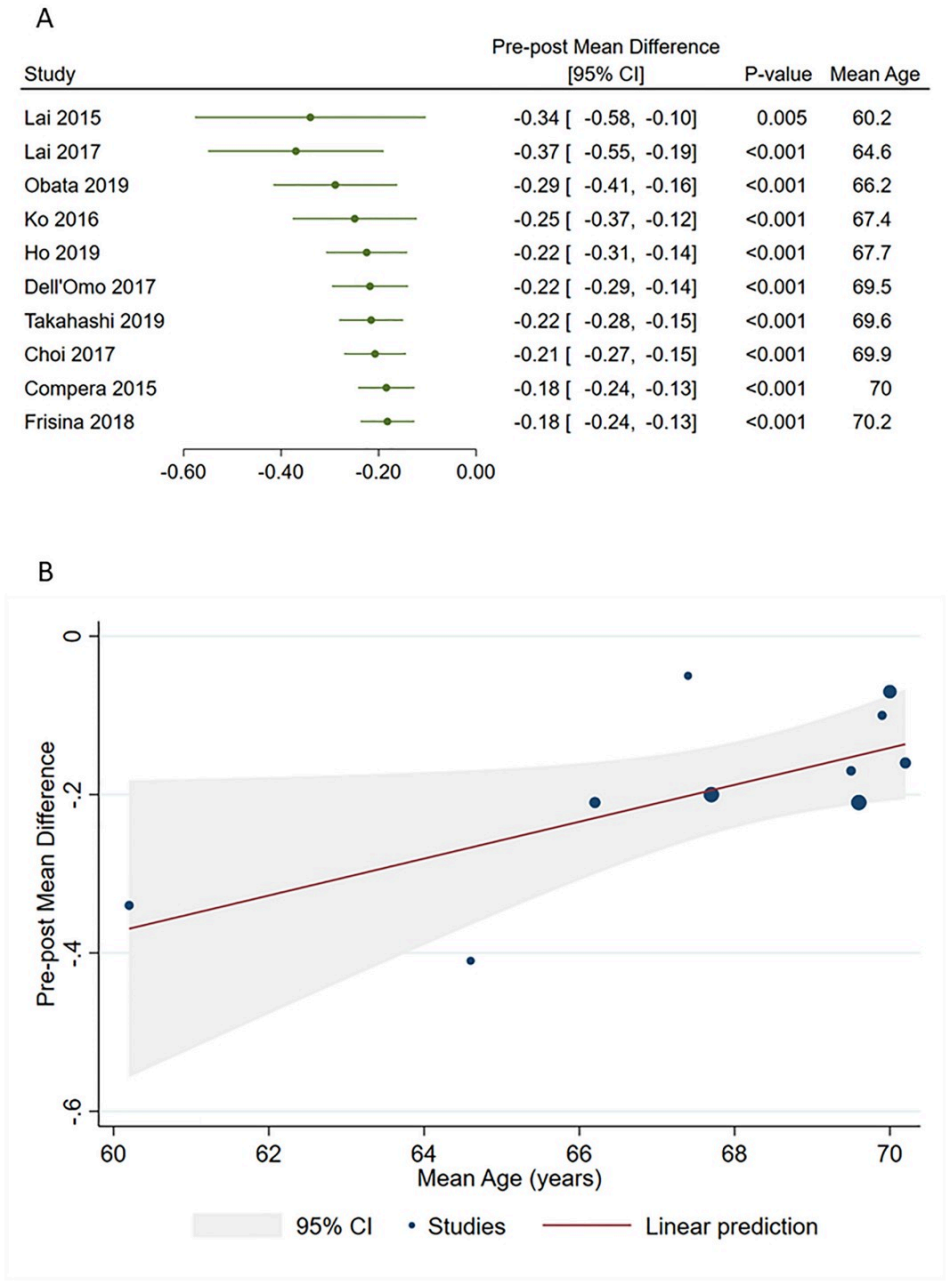
Cumulative meta-analysis (A) and meta-regression (B) showing relationship between patient age and pre-post mean difference in BCVA in the degenerative LMH and LHEP group.

**Fig 5:**
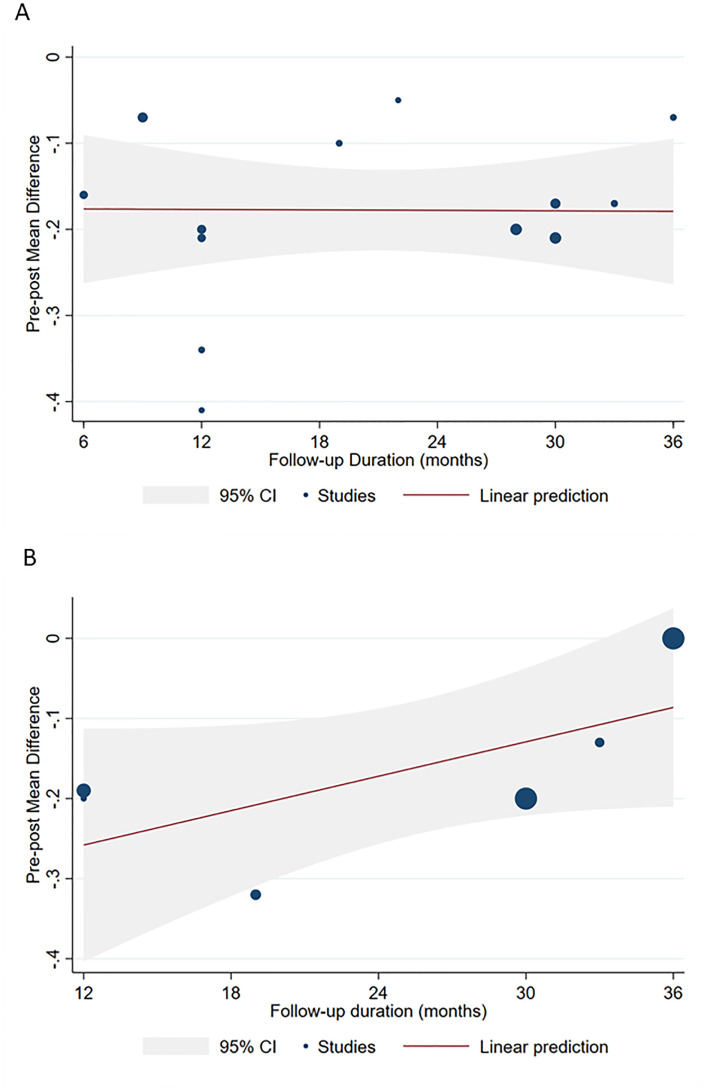
Meta-regression showing no significant effect of follow-up duration on pre-post mean difference in BCVA in the degenerative LMH and LHEP group (A) and in tractional LMHs (B).

We next sought to explore possible sources of heterogeneity by applying several meta-regressions. However, no relationship with mean age (p = 0.128), follow-up duration (p = 0.127), baseline BCVA (p = 0.239), or proportion of patients with LHEP (p = 0.456) was evident in the overall analysis. Instead, we noted a relationship between age and post-operative BCVA improvement among patients with degenerative LMH, as indicated by cumulative forest plot and bubble plot in Fig 4. Specifically, post-operative improvement declined with increasing age of patients with degenerative LMH (β = 0.02; 95%CI = 0.01, 0.05; p = 0.040), however, this was not found in the tractional LMH group. In the tractional LMH group, we instead observed a decline in post-operative visual acuity improvement with increasing follow-up duration. Yet, this apparent relationship was a trend with no statistical significance; in the degenerative LMH and LHEP group, no influence of follow-up duration was shown (Fig 5). None of the other moderators significantly affected post-operative BCVA improvement in patients with degenerative or tractional LMH.

### Incidence rate of full-thickness macular hole

Overall, the incidence rate of FTMH was low (0.1 events per ten person-years; 95%CI = 0, 0.2). However, we observed that it was higher in patients in the degenerative LMH and LHEP group than in those with tractional LMHs (0.3 events per ten person-years; 95%CI = 0, 0.6; versus 0 events per ten person-years; 95%CI = 0, 0; p = 0.002). We also noted significant heterogeneity in the whole analysis (p<0.01 for Q-statistics and I^2^ = 56.4%) and across studies in the degenerative LMH and LHEP group (p<0.01 for Q-statistics and I^2^ = 60.5%).

## Discussion

This systematic review and meta-analysis regarding the outcomes of primary PPV for LMHs, revealed a significant visual acuity improvement in both LMH subtypes, with a higher post-operative incidence rate of FTMH in degenerative LMH.

In 2016, Govetto *et al*. [3] redefined the concept of lamellar macular hole and, on the basis of OCT, classified LMHs into two clinical entities, degenerative and tractional, according to different diagnostic criteria. Specifically, degenerative LMHs were characterized by a ‘‘top hat” appearance with round-edged intraretinal cavitation, ellipsoid layer defect, presence of epiretinal proliferation, and a central retinal bump; tractional LMHs were characterized by a ‘‘moustache” appearance with a schitic sharp-edged intraretinal split, intact ellipsoid layer, presence of tractional epiretinal membranes, and intraretinal cystoid spaces.

This classification has been recently revised by a panel of international experts [21]. Tractional lamellar macular hole has been renamed "ERM foveoschisis", an entity that must show a contractile ERM as a mandatory criterion for the diagnosis, a key feature for differential diagnosis with degenerative or "true" LMH. This is in line with Govetto’s findings, which reported a contractile ERM in almost all cases (98%) of tractional LMH. In our study all eyes classified as tractional LMH were characterized by the presence of a contractile ERM. The definition of true LMH is very similar to that proposed by Govetto for degenerative LMH. Typical characteristics of true LMH are the presence of a foveal cavity with undermined edges, thinning of the fovea at its center or around the center, the presence of LHEP and a lower baseline visual acuity. Not surprisingly, our findings are in agreement with the above reported classification. In fact, in our study 93% of the eyes with degenerative LMHs presented with LHEP and 47% with ellipsoid defect. Mean baseline BCVA was worse, although not significantly, in the degenerative group compared to the tractional group, with an average difference of almost one line on the early treatment diabetic retinopathy study (ETDRS) chart between the two groups.

Parolini *et al*. were the first who conducted an immunohistochemical analysis of LHEP, showing this material is completely different from contractile ERM [2]. Lamellar hole associated epiretinal proliferation represents one of the main feature of degenerative LMHs [3].

Of note, we included in this review also studies that reported surgical outcomes of eyes with LHEP. Given the fact that LHEP is likely to be associated with degenerative LMHs, but less commonly could be associated with mixed LMHs, we contacted the authors of these studies to assess whether their cohort could be classified as degenerative LMHs according to Govetto’s definition [3]. On the one hand, Dell’Omo *et al*. [5], Parolini *et al*. [2], and Frisina *et al*. [9] confirmed that and their cohort were included into the degenerative LMH group. On the other hand, the author of 5 studies were unable to confirm the precise nature of LMHs and their cohort were included into the LHEP group [7, 10–13]. This clustering we adopted allowed us to conduct sensitivity analyses that excluded the LHEP group, reducing the risk of bias related to misclassification issues and providing, as a result, an evidence of higher quality. Additionally, we sought clarification about the presence of ERM in 8 eyes classified as degenerative by Dell’Omo *et al*. [5]. The authors confirmed that those eyes were affected by degenerative LMHs according to Govetto’s classification [3] and that they presented only focal trace of ERM which did not involve the fovea and did not cause traction. It must be noted that identifying the presence of trace amounts of standard ERM in eyes with degenerative LMH may be difficult and easily underestimated if the OCT scans do not encompass the entire macular area or if few OCT scans are made.

The management of lamellar macular holes has long been discussed. In the past, surgical treatment for LMHs was not a primary choice since LMHs were considered a stable clinical condition [22, 23] and surgery did not seem to ensure a functional improvement [24].

Thereafter, new evidence showed that observation could be associated with visual and anatomical worsening [25], while encouraging visual outcomes were reported following vitrectomy [26, 27]. This has supported a shift toward surgery rather than observation in the management of LMHs.

Following the introduction of Govetto’s classification, more attention has been given to the issue whether both degenerative and lamellar subtypes could benefit from surgical treatment.

Some authors reported an improvement in visual acuity after vitrectomy for both forms of lamellar macular hole [4, 5]. Conversely, other authors claimed an increase in visual acuity only in tractional LMHs, while no functional gain was found in degenerative LMHs [6, 7]. Additionally, higher rate of post-operative full-thickness macular hole has been reported after vitrectomy for degenerative LMHs compared to tractional ones. Hence, the management of degenerative LMHs appears to be controversial.

With regards to the visual outcome, the present meta-analysis demonstrated a comparable visual improvement after surgery in both the group of degenerative LMHs and LHEP eyes and the group of tractional LMHs. This result was confirmed by a sensitivity analysis that excluded the LHEP group, showing comparable visual outcomes between degenerative and tractional LMHs. This finding is of great clinical relevance because it can justify surgical treatment also in degenerative subtype. Of note, in the overall analysis we also included results from three studies that used modified peeling techniques for the treatment of degenerative LMHs. Since these innovative techniques were supposed to provide better outcomes than conventional ERM and ILM peeling, we decided to conduct a further sensitivity analysis excluding data from the cohorts treated with modified techniques [9, 12, 20], to verify whether traditional surgery still ensured comparable visual outcomes in both subtypes of LMHs. Indeed, this subgroup analysis confirmed that postoperative visual gain in degenerative and LHEP group was similar to the one in tractional group.

Interestingly, meta-regression analyses revealed a decline in postoperative visual acuity with increasing age in the degenerative LMH and LHEP group (Fig 4). This finding keeps with the clinical and pathogenetical characteristics of degenerative LMHs, which, even not well understood, collocate this entity within a degenerative process, given the damage to outer retinal structures. Like other degenerative process, older age and longer disease history could be associated with a poorer outcome [28, 29]. According to meta-regression analyses, follow-up length did not show any significant influence on the visual outcome (Fig 5). This might suggest that the surgery might help to stop the progress of the degenerative process, which seems rather related to aging.

The secondary outcome we wanted to explore was the incidence of postoperative FTMH in the two subtypes of LMHs. The pooled rate of postoperative FTMH was significantly greater in degenerative LMH and LHEP group compared to the tractional LMH group, with 0.3 events per ten person-year in the former versus 0 events per ten person-year in the latter, respectively.

The onset of FTMH after degenerative lamellar hole surgery is not uncommon, being reported with an incidence rate ranging from 4.3% to 27.7% [2, 6, 18]. This complication represents a major concern for surgeons because its treatment requires a new surgical procedure, with consequent influence on the final visual outcome [30]. A possible explanation for such an outstanding difference in postoperative FTMH rate between degenerative and tractional LMHs could be related to the fact that the epiretinal membrane peeling maneuver is more complex when LHEP is present. This could result more traumatic for Muller cells [31] compared to conventional ERM peeling [32].

In attempt to reduce the incidence rate of postoperative FTMH, some authors described modified technique [9, 12, 20]. No case of postoperative FTMH was found in these cohorts. However, in our analysis it was not possible to make a direct comparison between conventional and modified techniques as the latter ones differ from each other.

The present study presents some limitations. First, all the studies included in the quantitative analysis were retrospective. Therefore, this could have been a source of bias. Secondly, the data of individual patients were not available and the quantitative analysis was conducted from tabulated data extracted from each study. However, meta-analysis studies present greater power and more accurate confidence intervals than individual studies [33, 34]. Ultimately, eligibility criteria could have been different amongst included studies. With regards to high myopia, 3 studies considered a myopic condition >6 diopters as an exclusion criterion [6, 18, 19]; two studies excluded eyes with a myopia >8 diopters [2, 5]; seven studies did not specifically describe high myopia as an exclusion criterion[4, 7, 9–13], but two of them reported a mean axial length < 24.5 mm [7, 10]. Only Takahashi *et al*. acknowledged the inclusion of 10 patients with an axial lengths ≥ 26 mm [20]. Additionally, the included studies may also show some variability in clinical and surgical characteristics. This is particularly relevant because non-comparative studies were included as well. As a result, significant heterogeneity was found among the included studies for all analyses except the one on visual outcome in the degenerative LMH subgroup. The evidence we provide could be, to some extent, limited by this issue. However, meta-regression analyses were conducted with the purpose to assess the influence of possible clinical variables on the outcomes. Furthermore, to reduce a possible bias related to misdiagnosis issues, we conducted sensitivity analyses excluding the LHEP group. This is supposed to improve the quality to the evidence we provide.

In conclusion, our meta-analysis showed that both degenerative and tractional LMHs could benefit from vitrectomy in terms of visual outcome. However, in the degenerative subtype surgery has a greater chance of being complicated by FTMHs. Surgeons and patients should be aware of this risk when making a management plan. Randomized clinical trials with a large sample size and based on the most recent classification of LMH are needed in order to confirm our findings and investigate possible risk factors associated with the development of postoperative FTMHs.

## Supporting information

S1 Table PRISMA checklist. (PDF)

S2 Table Newcastle-Ottawa scale score for non-randomized studies. (PDF)

S1 Fig Funnel plot for pre-post mean difference in best corrected visual acuity in overall population. (PDF)

S2 Fig Funnel plots for pre-post mean difference in best corrected visual acuity in different LMH subtypes. (PDF)

S3 Fig A forest plot showing pre-post mean difference in best corrected visual acuity in studies featuring traditional surgical technique. (PDF)
