# Supplementary material for: Primary vitrectomy for degenerative and tractional lamellar macular holes: A systematic review and meta-analysis
Source: PLoS One. 2021 Mar 5;16(3):e0246667. doi: 10.1371/journal.pone.0246667 (PMC7935291; doi:10.1371/journal.pone.0246667)
Supplement: S2 Table — (PDF) [file pone.0246667.s002.pdf]

S2 Table: Newcastle-Ottawa Scale score for non-randomized studies

	SELECTION (max 4 stars)	COMPARABILITY (max 2 stars)	EXPOSURE (max 3 stars)	*TOTAL SCORE*
**Parolini et al 2011**	3	1	2	6
**Lai et al 2015**	3	1	2	6
**Compera et al 2015**	3	1	3	7
**Ko et al 2016**	3	1	2	6
**Coassin et al 2017**	3	1	3	7
**Choi et al 2017**	3	1	2	6
**Lai et al 2017**	3	1	2	6
**Dell'Omo et al 2017**	3	1	3	7
**Frisina et al 2018**	3	1	3	7
**Figueroa et al 2018**	3	1	3	7
**Obata et al 2019**	3	1	3	7
**Takahashi et al 2019**	3	1	2	6
**Ho et al 2019**	3	1	2	6
