# Supplementary material for: Primary vitrectomy for degenerative and tractional lamellar macular holes: A systematic review and meta-analysis
Source: PLoS One. 2021 Mar 5;16(3):e0246667. doi: 10.1371/journal.pone.0246667 (PMC7935291; doi:10.1371/journal.pone.0246667)

S2 Figure: Funnel plots for pre-post Mean Difference in best corrected visual acuity in different LMH subtypes

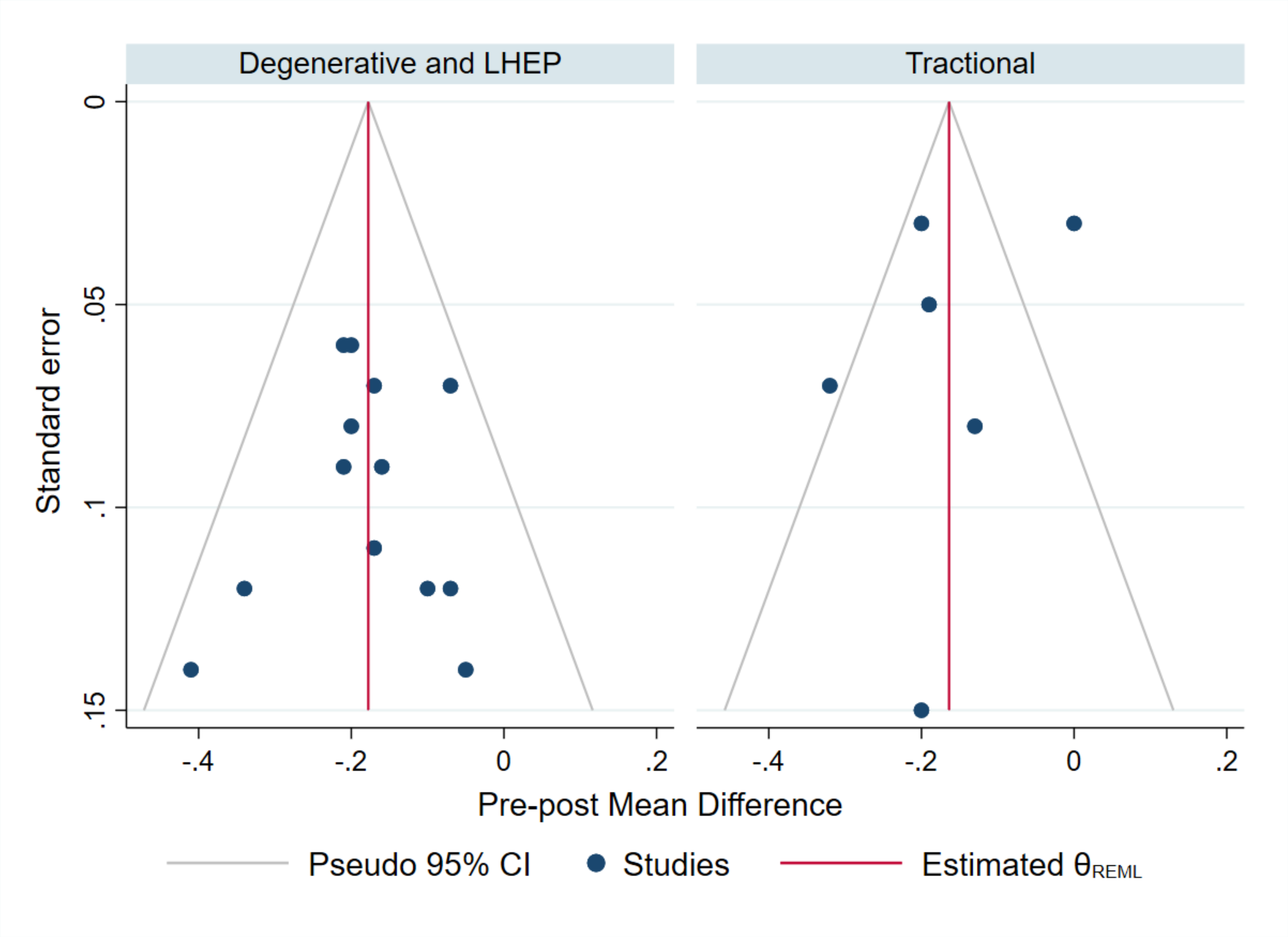

Supplement: S2 Fig — (PDF) [file pone.0246667.s004.pdf]
