# Supplementary material for: Primary vitrectomy for degenerative and tractional lamellar macular holes: A systematic review and meta-analysis
Source: PLoS One. 2021 Mar 5;16(3):e0246667. doi: 10.1371/journal.pone.0246667 (PMC7935291; doi:10.1371/journal.pone.0246667)

S3 Figure: A forest plot showing pre-post Mean Difference in best corrected visual acuity in studies featuring traditional surgical technique

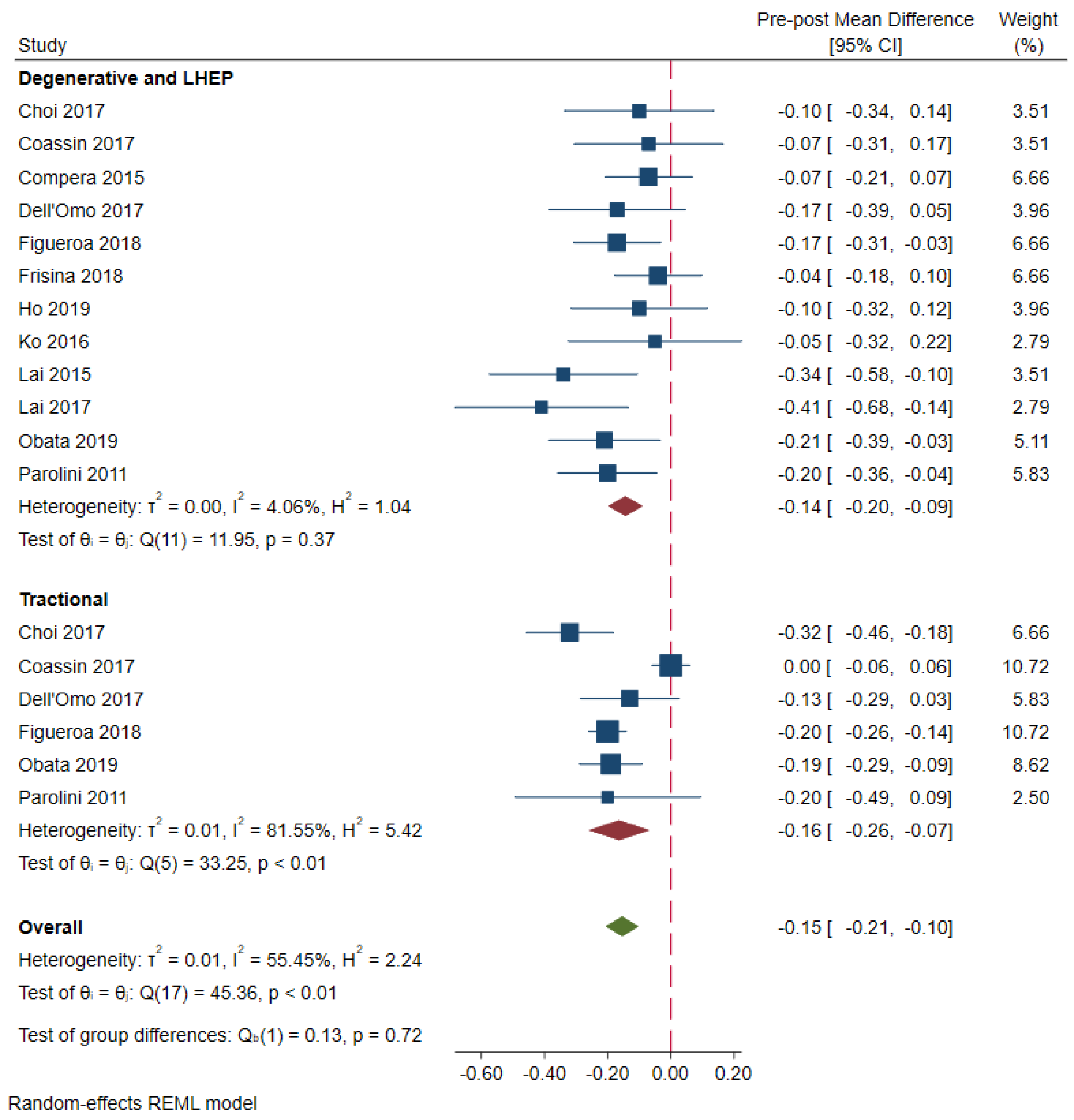

Supplement: S3 Fig — (PDF) [file pone.0246667.s005.pdf]
